# *Streptococcus agalactiae* Infection in Nile Tilapia (*Oreochromis niloticus*): A Review

**DOI:** 10.3390/biology13110914

**Published:** 2024-11-11

**Authors:** Ebtsam Sayed Hassan Abdallah, Walaa Gomaa Mohamed Metwally, Mootaz Ahmed Mohamed Abdel-Rahman, Marco Albano, Mahmoud Mostafa Mahmoud

**Affiliations:** 1 Aquatic Animal Medicine and Management Department, Faculty of Veterinary Medicine, Assiut University, Assiut 71529, Egypt; ebtsamsayed@aun.edu.eg (E.S.H.A.); mahmoud88@aun.edu.eg (M.M.M.); 2 Poultry and Fish Diseases Department, Faculty of Veterinary Medicine, Minia University, Minia 61519, Egypt; walaa_gomaa@mu.edu.eg; 3 Department of Behavior and Management of Animal Wealth, Faculty of Veterinary Medicine, Minia University, Minia 61519, Egypt; mootazabdelrahman@mu.edu.eg; 4 Department of Veterinary Sciences, University of Messina, Polo Universitario Dell’Annunziata, 98168 Messina, Italy

**Keywords:** teleost, Gram-positive cocci, biofilm formation, immunization, plant extracts

## Abstract

*Streptococcus agalactiae* (Group B Lancefield) has emerged as a significant pathogen affecting both humans and animals, including aquatic species. Infections caused by *S. agalactiae* are becoming a growing concern in aquaculture and have been reported globally in various freshwater and marine fish species, particularly those inhabiting warm water environments. This has led to numerous outbreaks with high morbidity and mortality in fish. Nile tilapia (*Oreochromis niloticus*), a member of the Cichlid family, is one of the severely affected fish species by *S. agalactiae.* The current study aims to focus on *S. agalactiae* infection in cultured *O. niloticus* with reference to its transmission and sources of infection; risk factors influencing GBS infection, disease clinical signs, lesions, and pathogenesis; *S. agalactiae* virulence factors; and how to diagnose, treat, control, and prevent infection including vaccination and herbal extract medication.

## Simple Summary

*Streptococcus agalactiae*, a significant pathogen affecting humans and aquatic species, is causing high morbidity and mortality in fish, particularly Nile tilapia (*Oreochromis niloticus*). This study focuses on *S. agalactiae* infection in cultured *O. niloticus*, examining transmission, sources, risk factors, clinical signs, pathogenesis, virulence factors, and methods for diagnosis, treatment, control, and prevention.

## 1. Introduction

Nile tilapia (*Oreochromis niloticus*) is a member of the Cichlidae family, which is the third largest family in the Osteichthyes class. Originally from Africa and the Middle East [1], tilapia has emerged as a significant aquatic species, with production occurring in approximately 100 nations worldwide [2]. Due to the growing commercialization and sustained expansion of the tilapia industry, it is considered the world’s most significant fish that is produced, second only to carp, but above the salmonid family [3]. *O. niloticus* is becoming a commercially important fish for aquaculture worldwide. China leads the world in tilapia production, with Egypt, Indonesia, and Thailand following behind [3]. This tropical species can reach sexual maturity in ponds at the age of 5–6 months and prefers to live in warm, shallow waters at approximately 25 °C [4,5]. Tilapia is well suited for large-scale aquaculture due to its rapid growth, simple reproduction, adaptability to feeding, and resilience to unfavorable water conditions [6]. However, its resistance to pathogens has been questioned [7,8].

The susceptibility of *O. niloticus* to bacterial, viral, and parasitic illnesses, including *Flavobacterium columnare*, *Edwardsiella tarda*, *Aeromonas hydrophila*, Spring Viremia of Carp virus, *Ichthyophthirius multifiliis*, *Trichodina* sp., *Gyrodactylus niloticus*, *Acanthogyrus tilapiae*, and *Lamproglena monodi* is well documented [7,9,10,11]. *O. niloticus* is particularly vulnerable to streptococcosis, which is the name of the disease itself rather than the genus causing it. Currently, 190 species of this genus have been reported [12]. Streptococcosis is caused by *S. agalactiae*, *S. iniae*, *S. parauberis*, and *Lactococcus garvieae* in warm water [13,14,15]. However, in colder temperate waters, *L. piscium* and *Vagococcus salmoninarum* are the causative agents [16]. The infectious agents of streptococcosis in warm water can spread to humans, terrestrial animals, and aquatic animals, globally, causing illness [15,17].

*Streptococcus agalactiae (S. difficilis*) is Lancefield’s Group B *Streptococcus*, known as GBS based on the presence and type of surface antigen [18]. The serotypes of GBS strains are assessed based on a capsular polysaccharide antigen. To date, GBS has been classified into 10 distinct serotypes, namely, Ia, Ib, and II-IX [19,20]. Among them, serotypes Ia, Ib, II, and III are the most prevalent in tilapia infections.

The bacterium is characterized by spherical or ovoid cells that are facultatively anaerobic, Gram-positive, oxidase-negative, catalase-negative, non-motile, and non-spore-forming with a 0.5–2.0 µm diameter. They are organized in pairs or short chains and require rich conditions for growth. They may also produce orange or yellow pigments [21]. GBS grows at temperatures between 25 °C and 45 °C [22]. Certain strains of GBS have a fermentative metabolism that primarily produces lactic acid as a byproduct of carbohydrate metabolism. Fish GBS strains cannot ferment sorbitol, mannose, and xylulose, nor can they hydrolyze urea and starch. However, GBS strains can ferment ribose and hydrolyze hippurate. The Voges–Proskauer reaction is positive, while the pyrrolidonyl arylamidase reaction is negative. Other biochemical parameters, such as the hydrolysis of arginine and aesculin, or the fermentation of trehalose, lactose, and inulin, vary depending on the strain being evaluated [23,24]. GBS can produce hemolysins, with strains classified as α-, β- [25,26], or nonhemolytic [22]. The CAMP reaction is positive only in hemolytic isolates [17,27,28]. However, fish-adapted GBS strains have also been described as nonhemolytic [29].

It has been recognized as a causative agent of diseases since the 19th century and has had significant impacts on aquaculture production, leading to substantial mortality and financial loss [26,30,31]. It is now clear that a variety of factors, such as environmental conditions and the presence of specific viruses and fungi, lead to outbreaks of tilapia streptococcosis [32,33]. Meningoencephalitis, caused by *S. agalactiae* infection, manifests as symptoms such as exophthalmia, corneal opacity, septicemia, and many abnormalities related to swimming [25,34].

Outbreaks of this causative agent of this zoonosis are causing alarm worldwide; infection rates have already reached 50%, and mortality rates have surpassed 95% [35]. The outbreaks of this infection occur mainly at temperatures above 26 °C and high stocking densities, and intensive production seems to increase the occurrence of this infection [27].

Streptococcal disease has been reported on all continents (Americas, Asia, Europe, Africa, and Australia) and in at least 15 countries [8]. Among the nearly 500 streptococcal isolates recovered from tilapia between 2001 and 2009, epidemiological investigations conducted in the major tilapia-producing regions of Asia and Latin America revealed that 82% of the isolates were identified as *S. agalactiae* and 18% as *S. iniae* [36]. Since 2009, *S. agalactiae* has accounted for more than 90% of the clinical bacterial isolates from infected tilapia in China [37]. Recently, *S. agalactiae* has been isolated from mass mortalities among cultured tilapia in Taiwan [38], Bangladesh [26], India [39], and Egypt [25].

Numerous freshwater and marine fish species are affected by GBS (Table 1), both in captivity and in the wild [40,41]. The most afflicted species are *O. niloticus* and its hybrids [25,27,33,42,43,44,45,46,47,48,49]. In addition to *O. niloticus*, other species affected include silver pomfret (*Pampus argenteus*), giant Queensland grouper (*Epinephelus lanceolatus*), sea bream (*Sparus auratus*), bighead carp (*Aristichthys nobilis*), and ya-fish (*Schizothorax prenanti*) [17,40,50,51,52,53].

## 2. Transmission and Sources of Infection

Numerous studies have examined how *Streptococcus* sp. spreads in a farming environment. The bacteria can infect fish directly through water, as evidenced by naturally occurring illnesses on farms. Factors such as minor wounds, abrasions, or external injuries to the fish´s skin, fins, or scales, as well as crowded or intense culture conditions, increase the likelihood of infection and disease [17,61]. Transmission of GBS also occurs through cannibalism of dead or moribund fish and indirect contact with bacteria in the water, allowing the disease to gradually spread in different production systems [62]. It was shown that *S. agalactiae* were released in infected *O. niloticus* feces [63], where they could survive in sterile freshwater incubated at 35 °C, 28 °C, and 15 °C for extended periods of time, reaching 80, 160, and 160 days post-inoculation [25], and infect nearby fish populations through the fecal–oral route.

The primary method by which a bacterial infection enters a system is through the introduction of new batches of fish into farms [13]. Additionally, fish can carry GBS without exhibiting symptoms, making them a significant source of infection for the epidemiological dynamics of streptococcosis [63]. Diseased or carrier fish typically release GBS through their gills, mucus, and feces [47,63], infecting the remaining healthy fish on farms. However, it is unknown how long the agent remains in the water after being eliminated from an infected fish. Some studies have indicated that the primary route of entry for GBS in fish is through the ingestion of contaminated water via the gastrointestinal tract [42] or infected fish [40].

Recently, *S. agalactiae* has been identified in infected tilapia during natural outbreaks and is pathogenic to fish through various experimental methods. Four infection methods have been documented: gill inoculation, injection, immersion bath, and cohabitation [25,27,34,42]. While intraperitoneal injections are commonly used in experimental infections, this method does not imitate natural conditions as bacteria must pass through all the natural barriers present in aquatic hosts to induce disease. Clinical symptoms were observed 24 h after infection in fish injected with *S. agalactiae* inoculum at a dilution ranged from 10^1^ to 10^8^ CFU/mL, with the first deaths occurring 72 h later [27]. In the cohabitation test, healthy fish began showing symptoms 24–72 h after contact with infected fish, and a 100% mortality rate was confirmed after 10 days [26,27]. The possibility of transmission through water-borne exposure is a concern, with the death rate being higher compared to injection challenges [25]. Gill inoculation has shown that gill tissue is a significant site for *S. agalactiae* infection in fish, resulting in a 33% mortality rate [27]. Furthermore, additional exposure methods for streptococcal infection in fish are used in experimental challenge investigations that can infect healthy fish. These methods include intramuscular injections, bathing, oral administration of food containing bacteria, plastic catheter or gavage, and nares inoculation [17,25,26,42,64,65,66,67]. Moreover, vertical transmission of *S. agalactiae* has already been demonstrated. Pradeep et al. [68] reported finding *S. agalactiae* in the testicles and gonads of tilapia breeders, as well as in 10- and 30-day-old larvae from breeders positive for the disease. This indicates a high potential for gamete transfer of the bacteria in tilapia broodstock [68].

## 3. Risk Factors Influencing GBS Infection

**Figure 1:**
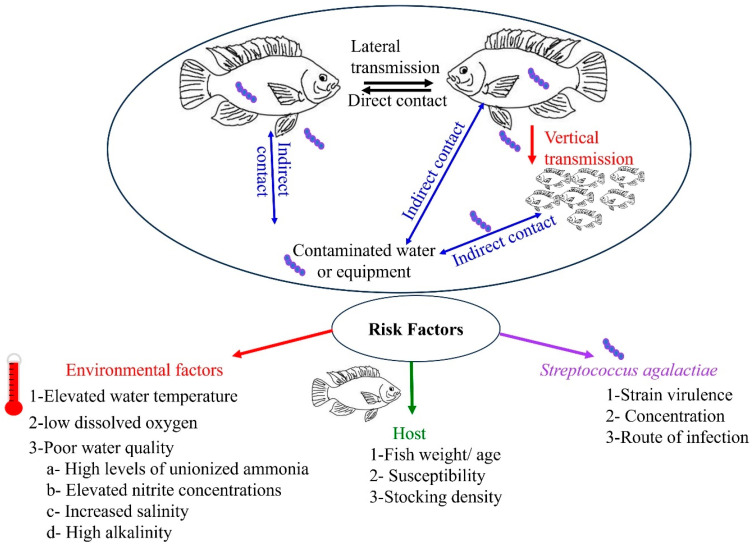
Illustration of *Streptococcus agalactiae* transmission, sources of infection, and risk factors influencing GBS infection.

In all bacterial fish diseases, the surrounding environmental conditions play a crucial role in affecting the uptake, colonization, and establishment of the diseases within susceptible fish species (Figure 1). Fish rely on their environment to maintain homeostasis, so suboptimal or variable conditions can have a significant impact. Few studies have documented the various environmental conditions that contribute to the establishment of *S. agalactiae* infection in tilapia. Factors such as high water temperatures (above 27 °C), low dissolved oxygen (DO) levels, high stocking density, intensive husbandry practices, unfavorable environmental conditions such as high ammonia, and fish weight, and/or age, among others, are believed to promote this disease. GBS outbreaks involving Nile tilapia have been associated with high mortality rates [25,27,57,69]. These factors have a direct and continuous effect on fish population health over time.

Elevated water temperature is considered a stressor that can increase the vulnerability of tilapia to *S. agalactiae* by promoting bacterial growth. The rate of bacterial multiplication and the production of virulence factors, both influenced by water temperature, can impact the severity of the disease. Therefore, a single environmental factor, like water temperature, can affect the progression of the disease, impacting both the host and the bacteria, and potentially heightening the vulnerability of individual fish [49]. This variability may explain the discrepancies in mortality rates observed during different clinical outbreaks.

Furthermore, environmental stressors and factors contributing to suboptimal water quality, such as high levels of unionized ammonia (≥0.02 mg/L) [65,70], elevated nitrite concentrations [57], increased salinity [39], alkaline water (pH > 8) [57], and high stocking densities [71], contribute to the occurrence of *S. agalactiae* outbreaks in tilapia species. These factors are often associated with intensive aquaculture practices and have been shown to induce stress responses in fish, compromising their immune systems.

Fish weight and/or age have been suggested as potentially significant factors influencing the development of *S. agalactiae* infections in farmed tilapia [72]. A random sample prevalence investigation revealed that the weight and/or age of the fish are crucial factors that predispose tilapia to outbreaks of *S. agalactiae* infection [8,27,72,73,74,75]. Infection is commonly observed in fish weighing less than 50 g, but it predominantly affects adult fish in the growth stage, with an average weight of 500 g [27].

Subsequent research has delved further into the role of *S. agalactiae* and host susceptibility in the initiation of infections in fish. Factors such as the specific bacterial strain or virulence expression, bacterial concentration, fish species, individual fish responses, infection routes, stock density, fluctuating environmental conditions, management practices, and other factors related to multiple coinfections have been identified as the primary influencing factors that affect the severity of infections [39,57].

## 4. Pathogenesis

The pathogenesis of *S. agalactiae* infection in tilapia has not been clearly described or understood. It is well established that crossing the blood–brain barrier is a crucial stage in the pathogenesis of GBS in humans; however, few studies have examined the function of genes involved in this process [76,77]. In fish, *S. agalactiae* can penetrate the blood–brain barrier, leading to meningoencephalitis [78].

In naturally infected fish, the first signs of pathological alterations are seen in the blood vessels. Bacterial colonies and exotoxins are present in tissue lesions in the liver, spleen, kidney, and brain [55,74,79]. The bacteria enter the bloodstream through local necrosis, internalize, and proliferate within macrophages [17,80], especially those of the spleen [34]. The infected cells either burst or undergo apoptosis, most likely due to the bacteria’s pathogenic mechanisms. This releases the bacteria within the organ, making it easier for them to spread to other organs through the blood (bacteremia) [34]. Another effect is that the immune response is less effective because apoptotic macrophages do not fulfill their function, and the presence of apoptotic bodies does not trigger an inflammatory response, unlike simple macrophage rupturing. This would allow new phagocytes to easily recognize bacterial cells. Macrophages may serve as a vehicle for *S. agalactiae*, allowing the bacterium to breach the blood–brain barrier, access the central nervous system, and spread more easily to other organs and tissues causing bacterial septicemia [40]. Disease initiation occurs when the host immune system fails to eliminate bacteria through phagocytosis. In *O. niloticus* tissues, the number of *S. agalactiae* copies peaked 24 h post-infection, with the bacteria primarily located in the blood in three different forms: freely dispersed without adhering to any structures, phagocytized by either phagocytes (primarily macrophages) or a small number of red blood cells, and adhered to the inner wall of blood vessels [81]. Moreover, *S. agalactiae* shows a preference for epithelial cells and can survive and multiply intracellularly after being phagocytosed by macrophages, where macrophages may serve as pathogen carriers or “Trojan horses” to facilitate immune evasion and disrupt the blood–brain barrier (BBB), leading to meningitis in *O. niloticus* [81]. Similarly, without the aid of complement or antibodies, murine macrophages were able to phagocytose *S. agalactiae* in large amounts in a dose-dependent manner using the phagocytosis assay [82]. GBS was highly effective in entering macrophages and remained intracellularly for more than 24 h [82].

## 5. Virulence Factors of GBS

### 5.1. Capsular Polysaccharides (CPSs)

Ten separate serotypes (Ia, Ib-IX) of the *S. agalactiae* group of bacteria have been discovered based on unique biochemical structures of the polysaccharide capsule (CPS) [83]. Currently, serotypes 1a, 1b, and III of *S. agalactiae* are thought to be the main strains influencing the global tilapia fish farming sector [58]. CPSs are pathogenic components commonly found in *Streptococcus* serotypes and are typically used for strain typing. It has been established that CPSs increase the severity of illness [84]. Experiments have shown that bacterial cells without the ability to produce CPSs lose their virulence in a neonatal rat model of lethal GBS infection [85,86]. It is now understood that CPSs can prevent complement factor C3b from aggregating and thus prevent host cells from being phagocytosed and killed [87,88]. Sialylated CPSs are similar to cell surface carbohydrate epitopes, reducing host immune recognition [89]. Further, the CAMP Factor (co-hemolysin) is encoded by the cfb gene. It is an extracellular protein of 23.5 kDa [90] that enhances GBS pathogenesis [91,92]. In essence, the pathogenic effects of CAMP involve its oligomerization, which aids in creating specific pores in host membranes, and its binding to proteins anchored by glycosylphosphatidylinositol (GPI), potentially leading to cell lysis [93]. Two research teams have recently determined the structure of CAMP, shedding light on its perforating activity [94,95]. Additionally, Podbielski et al. [96] demonstrated that a full-sized recombinant CAMP exerts cohemolytic effects.

### 5.2. HylB Gene

Using the selective capture of transcribed sequences (SCOTS) technique, Guo et al. [82] found that interaction with murine macrophages increased the expression of the hylB gene, which encodes the S. agalactiae hyaluronidase (HAase). Hyaluronic acid (HA) is broken down by the endoglycosidase hyaluronidase (HAase), which cleaves glycosaminoglycan chains [97]. Hyl may be a crucial element in facilitating the spread of pathogens from an initial site of infection, as it is a significant component of the ground material of the majority of connective tissues, especially the skin. By secreting HAase, which particularly hydrolyses the host cell wall component of hyaluronic acid into unsaturated disaccharide units as the end result, GBS facilitates its invasion of hosts. Additionally, it is now evident that GBS counteracts host immunological responses by using HAase [98]. The secreted HAase from GBS breaks down proinflammatory HA fragments into their component disaccharides, preventing the host’s TLR2/4 signaling responses. Normally, a host can react quickly by producing hyaluronan (HA) polymers, from which tiny fragments eventually combine with Toll-like receptors (TLRs) to elicit inflammatory responses [98].

### 5.3. Cel-EIIB

The GBS phosphotransferase system (PTS) has been shown to control bacterial pathogenicity by phosphorylating sugar substrates such as lactose, fructose, cellobiose, mannose, and sorbose [99]. Many GBS serotypes have high levels of cellobiose-PTS (cel-PTS) expression. The cel-PTS component cel-EIIB is expressed at different levels in low- and high-virulence GBS [100,101]. When compared to the wild-type GBS strain, the cel-EIIB knockout strain was found to have a decreased ability to utilize cellobiose, as well as a significantly lower ability to form biofilms [102]. Furthermore, the cel-EIIB knockout considerably decreased the effectiveness of invasion and colonization and resulted in a 20% reduction in the cumulative mortality of *O. niloticus* following GBS infection [102].

### 5.4. Cellobiose-PTS (Cel-PTS)

It is expressed in various serotypes of GBS, and strains lacking cel-PTS genetically have reduced colonization ability and virulence [102].

### 5.5. Quorum Sensing (QS) System

The quorum sensing (QS) system is a coordinated method of controlling gene expression that stimulates bacterial communication and group activity [103]. The *LuxS* gene encodes S-ribosyl homocysteinase, which catalyzes the production of the QS signaling molecule autoinducer 2 (AI-2), a furanosyl borate diester. *LuxS* is conserved across GBS serotypes and is widely expressed [104]. Ma et al. [105] showed that a mutant strain lacking *LuxS* had over a 30-fold decrease in acid resistance and was defective in quorum sensing compared to the wild-type strain. Additionally, cell adherence was reduced in the mutant strain. A study on tilapia demonstrated a significant decrease in infection levels when *LuxS* was restored to the *LuxS* mutant strain, leading to a restoration of hypervirulence [105].

### 5.6. Biofilm Formation

To enhance its ability to colonize and survive in its host, GBS can create three-dimensional structures like biofilms. Environmental factors greatly impact this process. It is widely recognized that bacterial biofilms are crucial for virulence and can result in long-lasting infections. Various adhesins have been found to contribute to the formation of GBS biofilm-like structures, including the protein components of pili that extend from the bacterial surface. Interestingly, antibodies that target pilus proteins have been shown to prevent biofilm formation [106,107]. Recently, it was discovered that every *S. agalactiae* isolate produced biofilms [25,108]. Of the examined isolates, 67 (72.8%) were classified as strong biofilm producers, 20 (21.7%) as moderate biofilm producers, and 5 (5.43%) as weak biofilm producers [108]. Additionally, Abdallah et al. [25] revealed that all seven isolates developed biofilms after 48 h of incubation at 28 °C. Six out of the seven isolates were moderate biofilm producers with optical density (OD) values up to four times higher than the negative control. Only one isolate was identified as a strong biofilm producer with an OD value greater than four times that of the negative control.

## 6. Methods of Diagnosis

**Figure 2:**
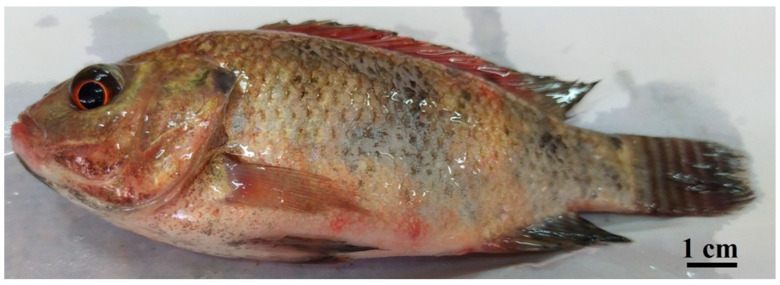
Septicemic picture of natural *Streptococcus agalactiae* infection on cultured Nile tilapia (*Oreochromis niloticus*). Photo was photographed by Dr. Ebtsam S. H. Abdallah.

The same guidelines that apply to other vertebrate animals are used to diagnose bacterial infections in fish species. When a disease outbreak occurs, the best course of action would be to collect information about the outbreak’s history. This includes obtaining fish tissues from sick fish that exhibit obvious disease symptoms as well as healthy fish from the same location (pond, cage, etc.). The diagnosis of *S. agalactiae* infection in tilapia should be based on standard clinical signs, lesions, and the presence of Gram-positive coccus bacteria isolated from the internal organs of the affected fish. GBS can infiltrate and reproduce in various organs of affected fish, leading to septicemic illness. However, it is believed that the brain tissue is the primary target, as the bacterium causes meningoencephalitis, resulting in clinical signs such as nervousness, aberrant behavior disorientation, and erratic swimming in spiraling and spinning. Additional clinical symptoms that may be observed in infected fish include anorexia, lethargy, melanosis, dorsal rigidity, a C-shaped body curvature, altered body curvature, vertebral malformation, corneal opacity, unilateral or bilateral exophthalmia, commonly known as “pop-eye”, peri-orbital or intraocular hemorrhage, diffuse hemorrhage in the skin (Figure 2) and musculoskeletal tissue, and ascites [17,25,26,27,40,54,56,61,72,73,109,110,111,112,113]. Not every infected fish exhibits these clinical indications, and affected fish show no obvious signs before sudden death [35,48,49,65,80]. Necropsy revealed yellow or dark red nodules in the muscle tissue, a pale liver, hepatomegaly, splenomegaly, clouded meninges or cerebrospinal fluid, and the accumulation of serosanguinous fluid in the fish´s abdominal cavity [40,45].

Histopathological changes in systemic streptococcosis have been observed in many organs and tissues, characterized by a mixed mononuclear inflammatory response. These changes are particularly prominent in the brain, heart, and eyes [34,74,114]. Diffuse granulomatous meningitis, multifocal branchiitis characterized by the proliferation and hyperplasia of gill lamellar epithelium and fusion of adjacent gill lamellae, blood vessel congestion and dilation with bacteria circulating within macrophages, endophthalmos and choroiditis with a varying degree of granulomatous inflammation in the tissues, keratitis with ulcers in the cornea and edema between adjacent stromal layers, pancreatitis, peritonitis, nodular granulomatous splenitis with congestion and hemorrhage of spleen tissue, granulomatous epicarditis, pericarditis, myocarditis, endocarditis, interstitial nephritis, hepatitis, gastritis, enteritis with the presence of bacteria in the intestinal lumen and lamina propria, subsequent degeneration and necrosis of ellipsoids and depletion of the white pulp, myositis of skeletal muscle with granulomatous nodules of central necrosis and accumulation of bacteria within the granulomas, and finally, ulcerative and hyperemic dermatitis [34,45,109,114].

Due to the wide range of vulnerable hosts and universal clinical manifestations confirmed in fish infections caused by various *Streptococcus* species, laboratory diagnosis is essential for identifying the specific etiological agent responsible for outbreaks [115]. Therefore, the diagnosis of GBS is based on the isolation and identification of microorganisms. Fish that are moribund can be collected and promptly delivered to diagnostic labs on ice [116]. It is best detected by sampling nerve tissue or highly vascularized organs, such as the kidney, liver, and spleen, which are involved in immunological processes [25,26,117]. The intestine, heart, and eyes are also used to diagnose this disease [17,34,42,118]. Additionally, venipuncture and kidney aspiration are safe, feasible, and non-lethal sampling techniques for obtaining blood and kidney samples from *O. niloticus* to diagnose GBS infection [63].

In bacteriology, tissue swabs obtained aseptically are streaked on standard culture media such as blood agar, Todd–Hewitt agar (THA), brain heart infusion (BHI) agar, and tryptic soy agar (TSA) [17,25,27]. Selective media like streptococcal selective agar [25], Columbia blood agar, and chromID Strepto B agar [48,119] can also be used. Additionally, samples can be enriched in Lim broth or Granada biphasic broth, followed by posterior subculturing in culture media to select GBS in suspected infection cases [63,120]. After 48–72 h of incubation at 28 °C, bacteria were identified based on assessing the features of bacterial colonies, observing cell morphology under a light microscope (using Gram-stain), determining the type of hemolysis, analyzing the structural pattern of the capsular antigen (known as the Lancefield group antigen), and conducting biochemical assays like catalase and oxidase [62]. The use of commercial kits, such as RAPID32 and API20 Strep, for the phenotypic characterization of GBS has shown good applicability, accuracy, and time savings [27]. However, misidentifications or a lack of species-level resolution may occur when isolating and characterizing bacteria using biochemical and phenotypical testing [121]. Therefore, complementary molecular techniques are necessary for accurate diagnosis.

Several strategies, including PCR amplification and *16S rRNA* gene sequencing, can be used for the molecular biology detection of GBS [27]. Other methods include species-specific PCR [122], species-specific qPCR [123], multiplex PCR [117], nested PCR [76], loop-mediated isothermal amplification (LAMP) [124], and matrix-assisted laser desorption ionization (MALDI)–time of flight (TOF) mass spectrometry [121]. Among these methods, GBS-specific PCR and *16S rRNA* gene sequencing have been extensively used for a definitive diagnosis of the disease. By performing PCR amplification of the universal *16S rRNA* gene in bacterial pathogens followed by sequencing of the obtained amplicons, it is possible to compare the DNA sequence of an identified isolate with others deposited in public databases like the NCBI (www.ncbi.nlm.nih.gov/BLAST accessed on 20 August 2024). Sequences that exhibit at least 97% similarity are considered to be the same species of bacteria [125].

Primers from the 16S–23S intergenic spacer regions (IGSs) were utilized for GBS-specific PCR. This method confirms GBS strains in questionable bacterial isolates from culture media, as no amplicons are formed during amplification from related *Streptococcus* species. A multiplex PCR approach was used to simultaneously detect Gram-positive fish pathogens such as GBS, *S. iniae*, *S. parauberis*, and *L. garvieae*. The results showed that this assay is a reliable tool for the fast and specific detection of GBS infection using both pure culture (detection limit = 250 to 125 cells) and fish tissues (detection limit = 12,000 cells/g) [115]. Similarly, nested PCR using the 16S–23S *rRNA* gene was performed for GBS identification in naturally infected fish and in frozen and paraffin wax-embedded tissues [75]. Nested PCR demonstrated a high sensitivity for GBS detection in these samples, with detection limits varying from 6.95 picograms to 1.58 femtogram for the DNA extracted from each sample [75].

Interestingly, the 16S–23S *rRNA* gene was also utilized to create a qPCR assay. This assay revealed that the tissues with the highest bacterial load following experimental infection were the brain (105 copies/mg tissue), eye (104 copies), spleen (104 copies), and kidney (104 copies). It provides a quick, sensitive, and accurate method to identify and precisely quantify GBS in fish tissues [123]. When comparing the frequency of GBS detection in various tissues of experimentally infected tilapia using the species-specific PCR method developed by Mata et al. [115] with the species-specific qPCR method described by Su et al. [123] and Tavares et al. [63], qPCR proved to be more sensitive than conventional PCR. It detected GBS in 95.3% of infected fish, whereas the detection rate of the other technique was only 51.1%.

The LAMP assay is a diagnostic technique that allows for the visual detection of pathogens when paired with a dye indicator. This method of GBS diagnosis was used to identify bacteria in the testes and ovaries, as well as other tissues of broodstock fish [68]. Additionally, the investigation and confirmation of the infection also included milt, eggs, and larvae from broodstock [68]. However, the MALDI-TOF method enables the identification of various bacterial species by comparing their peptide mass fingerprints with those of well-recognized GBS fish strains in the device database [121].

One crucial routine identification method for *S. agalactiae* is the serological detection of group-specific cell wall carbohydrate antigens. These group-specific C-carbohydrate antigens are detected using immunological GBS identification techniques, which include direct antigen detection, the latex aggregation or coagglutination test [26,126], and enzyme immunoassay [120,127]. Additionally, a quick 15 min GBS detection method utilized an overnight enrichment culture and immunochromatography approach that targets the Sip antigen, a surface immunogenic protein unique to GBS and frequently expressed in GBS strains of any serotype [126].

## 7. Treatment

The most common treatment strategy during a confirmed bacterial disease outbreak in farmed fish populations is to administer antibiotics. Typically, antibiotics are administered through the feed. Studies have shown that the majority of *S. agalactiae* strains are susceptible to a variety of antibiotics in various fish species [17,50,53,64,128]. Isolates of *S. agalactiae* recovered from *O. niloticus* are susceptible to various antimicrobial treatments. Variations in susceptibility and resistance to antibiotics within the same species of bacteria may result from differences in the serotypes and from frequent or improper use of chemotherapy. For example, using these medications in fish farms at inadequate concentrations or for insufficient periods [81,112].

The oral use of antibiotics such as amoxicillin, enrofloxacin, oxytetracycline, and florfenicol is utilized to treat GBS infection [129]. Some of these medications have demonstrated efficacy against fish GBS strains in vitro, as indicated in Table 2. The two primary techniques employed to assess GBS susceptibility to antibiotics in vitro are minimum inhibitory concentration (MIC) determination and disk diffusion assays [130]. Nevertheless, in addition to antimicrobial susceptibility, other factors that may impact the efficacy of treatment include the pharmacokinetic and pharmacodynamic properties of the antibiotic, the maximum plasma concentration, tissue distribution, and the dosage of the drug [131]. Antibiotics need to be administered to brain tissue since GBS causes meningoencephalitis in fish by crossing the blood–brain barrier. However, there is limited evidence available regarding the ability of antibiotics such as oxytetracycline and florfenicol to cross the blood–brain barrier. Decreased food consumption, with anorexia being one of the earliest physiological changes caused by GBS infection, is another issue related to the use of antibiotics. Antibiotic therapy is limited in that it treats the early stages of sickness and prevents the disease from occurring in healthy fish, but it does not cure fish that have clinical indications [130].

The effectiveness of oxytetracycline and florfenicol as treatments against GBS was assessed in vivo through trials involving the oral administration of these two antibiotics to *O. niloticus*. In the florfenicol trial, the normal antibiotic dose (10 mg kg^−1^) failed to control GBS infection in experimentally infected fish, resulting in a 90% mortality rate during the 10-day treatment period [133]. Conversely, doses of 20 and 40 mg/kg effectively suppressed the infection process over the same time frame. However, cumulative mortalities were observed in all treated groups 20 days after treatment initiation. This indicates that regardless of the dose administered, the medication was unable to halt the infection in fish, allowing the disease to spread to healthy fish in cohabitation experiments [133].

In the oxytetracycline trial, it was shown that compared to the control group (which did not receive antibiotic administration), the number of dead *O. niloticus* was much lower after the drug was administered (24 h before the experimental infection, 1 and 24 h post-infection). Nevertheless, the bacteria could be isolated again from the brain and kidney tissues of the fish that appeared to be in good condition across all treated groups following the experimentation period, indicating the carrier state of the infection [130]. Trials using florfenicol yielded similar results, indicating that either antibiotic may not effectively control the infection. This suggested that the pathogen could persist in living form within various fish tissues, ultimately leading to the bacterium’s persistence in fish farms.

Although synthetic and natural substances, such as herbs, have been shown to possess antibacterial properties in both in vivo and in vitro studies, their effectiveness can vary when used under field conditions. A key factor contributing to this variability is the inability of animals to respond to a therapeutic dose, likely due to the rapid onset of anorexia in diseased animals and the emergence of drug-resistant strains [134]. Additionally, concerns about drug residues and withdrawal times in farmed fish for human consumption, as well as the potential negative impact of antibiotics on the environment, further complicate the use of antibiotic therapy. Consequently, antibiotic therapy may not always be the most effective treatment option. However, these challenges can be addressed by improving stock density, water quality, the overall environment, and management practices. A combined strategy that addresses these factors is likely to be more successful in managing bacterial infections in fish. The use of medicinal herbs and other plants in aquaculture offers many advantages over the use of chemicals. These benefits include improved growth performance, antioxidant activity, physiological conditions, and welfare status [135]; antimicrobial [25]; and hepatoprotective effects [136]. Additionally, therapeutic plants are more cost-effective, readily available, and biodegradable compared to artificial pharmaceutical substances [137,138].

## 8. Prevention and Control

Standard preventive strategies to limit *S. agalactiae* infection in intensively farmed tilapia include improving environmental conditions and water quality, as well as reducing overcrowding. To lower the risk of disease outbreaks and reduce the transmission of pathogens, it is also important to avoid overfeeding, minimize unnecessary handling or transportation, and remove moribund and dead fish as soon as possible. Periodic tank cleaning and proper disinfection of all production units and utensils should be regularly conducted. Additionally, aquaculture could benefit from the utilization of probiotics, synthetic chemicals, herbal remedies, and nonspecific immunostimulants, alongside vaccinations, as methods for controlling streptococcosis [61,139,140].

In aquaculture, antibiotics have been used to control and eliminate pathogenic microbes [141]. Fish are often stressed in agricultural environments, leading to a decrease in the efficiency of their immune system [142]. As a result, antibiotic use in managing diseases in fish has become common due to their inability to escape bacterial colonization and infection [142,143]. However, frequent use of antibiotics often exposes a bacterial population to increased selective pressure, leading to the emergence of antibiotic resistance [143]. The region surrounding farming sites has higher than average concentrations of resistant bacteria and residual antibiotics [144]. In addition, horizontal gene transfer of resistance genes occurs between tolerant bacteria and other potentially more virulent pathogens [145]. If human pathogens acquire resistance determinants, it could have a detrimental impact on human health [146,147]. Additionally, the use of antibiotics has significantly decreased. In certain situations, they are ineffective at treating bacterial infections in fish [141,148]. In addition to their inefficiency, routines have been established to protect fish from bacterial diseases, leading to a reduction in the use of antibiotics [149,150].

Several experimental GBS vaccines have been developed and extensively reviewed by Liu et al. [36] and Miyabe et al. [151]. In aquaculture, both oral and immersion vaccinations are utilized for fish as they require less labor, are quicker, and are believed to be less stressful for the fish. On the other hand, injection vaccines can be given manually or with the help of semi-automated equipment.

Limited studies have been conducted on vaccinating tilapia against *S. agalactiae* infections. Eldar et al. [152], Pasnik et al. [153], Pretto-Giordano et al. [154], and [155] developed an injectable, modified killed *S. agalactiae* vaccine made of bacterial protein and entire cells to protect *O. niloticus* from streptococcosis. Experimental investigations have shown the effectiveness of this vaccination against infection in *O. niloticus*, with a relative percentage of survival (RPS) ranging from 49% to 100% [156].

Additionally, Evans et al. [157] and Evans et al. [158] demonstrated the efficacy of a formalin-killed *S. agalactiae* vaccine administered via intraperitoneal injection. The commercial AQUAVAC^®^ Strep Sa vaccine, developed by Merck Animal Health, has been available in many countries since 2011. It is an inactivated oil adjuvant vaccine that can stimulate active immunity against *S. agalactiae* biotype II (serotype Ib) and can be administered to fish weighing more than 15 g via injection. This vaccine has been tested in *O. niloticus* but is suitable for other fish species susceptible to *S. agalactiae* biotype II (serotype Ib). In their study, Fyrand and his colleagues used *S. agalactiae* serotype Ia that were originally isolated from three different *O. niloticus* farms located in North America, Central America, and Southeast Asia. They reported that regardless of the bacterial strain used for the challenge, *O. niloticus* were highly protected against cross-challenge when vaccinated with monovalent oil-adjuvanted vaccines containing *S. agalactiae* whole-cell antigen from distinct genetic groups. No significant difference in the level of protection was observed [159]. Additionally, the administration of a bivalent formalin-inactivated whole-cell vaccination against *S. agalactiae* serotypes Ia and III resulted in comparable levels of specific antibody production against both serotypes in *O. niloticus*, and at 30 days, the relative percentage of survival was considerably higher than that of the monovalent vaccine (*p* < 0.05) [160]. Moreover, upon conducting a farm trial across various regions of Thailand, the authors discovered that the bivalent vaccine was successful in increasing output yield by over 80% in every farm that was examined [160]. Nevertheless, additional research is needed to evaluate the relationship between genetic diversity, antigenic characteristics, and the ability to overcome heterologous challenges posed by this vaccination. Therefore, to more accurately assess the effectiveness of this commercial vaccine, it should be tested against different fish GBS strains, especially those with unique genotypes and serotypes.

As preventatives and control measures for a variety of fish diseases, alternative biocontrol techniques like bacteriophage therapy, phytodrugs, and probiotics are gaining more and more attention. Probiotics are useful as a preventative tool against bacterial diseases; however, there have been instances of antibiotic resistance [161] and interspecies genetic exchange in probiotic bacterial strains [162], which calls for ongoing safety monitoring. Because phytodrugs release exogenous chemicals into the marine environment, there have been reports of environmental damage [138]. The pace at which bioactive components are absorbed by fish and the toxicity of some chemicals to fish are two significant drawbacks [163]. Phage therapy in aquaculture has recently made significant advancements. It is now widely used due to its auto-dosing capacities, self-limiting nature, high specificity against its host, and safety in application. Unlike traditional antibiotics, phage therapy does not harm normal microflora or eukaryotic cells, making it a safe and environmentally friendly option [164]. One effective biocontrol agent that can be used to prevent and treat bacterial infections is bacteriophage. In 1969, Russel and colleagues isolated and described the first bacteriophage against *S. agalactiae* [165]. When employing bacteriophage HN48 to combat *S. agalactiae* infections in aquaculture, the results were encouraging [166]. Recently, potential lytic phages from *Myoviridae* and *Siphoviridae* morphotypes were discovered to combat *S. agalactiae* in Nile tilapia [164].

Herbal remedies have been found to effectively manage diseases in aquaculture. There is a growing body of research on the use of herbs to combat *S. agalactiae* in tilapia, as the demand for more environmentally friendly aquaculture practices increases. For instance, Abdallah et al. [25] reported that the ethanolic leaf extracts of nine medicinal plants demonstrated considerable antibacterial activities against the tested *S. agalactiae* strain with low minimum bactericidal concentrations (MBCs) and minimum inhibitory concentrations (MICs). The ethanolic leaf extracts from *Lantana camara* and *Aberia caffra* showed potent antibacterial activity with MBC values of 0.24 and 0.485 mg/mL, and MIC values of 0.12 and 0.24 mg/mL, respectively. Additionally, Borisutpeth et al. [167], Pirarat et al. [168], and Wongthai et al. [169] reported that four herb extracts—*Hibiscus sabdariffa*, *Cassia fistula*, *Citrus grandis* (*C. maximus*), and Red *Kwao Krua* (Butea superb Roxb.)—exhibited antibacterial activity in vitro against *S. agalactiae* isolated from diseased *O. niloticus*. Rattanachaikunsopon and Phumkhachorn [170] as well as Rattanachaikunsopon and Phumkhachorn [171] were observed to exhibit decreased mortality in *S. agalactiae*-infected Nile tilapia when fed a diet supplemented with *Andrographis paniculata* or *Cratoxylum formosum* extracts. Furthermore, the findings of Rattanachaikunsopon and Phumkhachorn [171] suggested that the aqueous extract of *C. formosum* could serve as an immunostimulant to hinder *S. agalactiae* infection. The results of the study showed that the innate immune responses of tilapia, such as phagocytic, lysozyme, and respiratory burst activities, were enhanced when an aqueous extract of *C. formosum* was included in their diet. Similarly, feeding *O. niloticus* under experimental conditions with dry extracts of rosemary (*Rosmarinus officinalis*) and *Pseuderatherum palatiferum* leaves significantly reduced mortality rates following infection with *S. agalactiae* [172,173]. In the trial aimed at treating antibiotic-resistant *S. agalactiae* in cultured Nile tilapia, fraxetin, a herbal medicine belonging to the coumarin derivative isolated from *Fraxinus rhynchophylla*, was used. This resulted in a significant decrease in the adhesion ability of *S. agalactiae* in a dose-dependent manner. Additionally, it reduced the mortality of tilapia infected with *S. agalactiae* to 46.67%. These findings suggest that fraxetin can offer significant protection to tilapia by inactivating the *S. agalactiae* transpeptidase enzyme Sortase A (SrtA), indicating that fraxetin is a novel inhibitor of *S. agalactiae* SrtA and a promising candidate for treating *S. agalactiae* infections in aquaculture [174].

Currently, there is a growing interest in using various synthetic chemicals and microorganisms in fish feeds to reduce *S. agalactiae* infection in *O. niloticus*. For instance, Samrongpan et al. [175] showcased the benefits of incorporating mannan oligosaccharide (MOS) into the diet of *O. niloticus* fry to improve growth and boost resistance to *S. agalactiae* disease. Ng et al. [176] found that red hybrid tilapia, when fed 0, 1, 2, or 3 g/kg of organic acid and exposed to 10^5^ CFU/mL of *S. agalactiae*, exhibited significantly higher survival rates ranging from 66.7% to 83.4% compared to the control group (41.7%). Probiotics have also been explored, with a study by Srisapoome et al. [177] showing that tilapia fed a diet enriched with *Bacillus pumilus* experienced lower mortality rates. These studies highlight the potential to enhance the resistance of *O. niloticus* to *S. agalactiae*-induced disease.

## 9. Limitations of Current Studies and Future Research Directions

The pathogenicity, modes of transmission, and effects on fish health have been the main topics of recent research on GBS in *O. niloticus*. Limitations, however, include the need for more thorough field research to comprehend the ecological dynamics and interactions within aquatic ecosystems as well as the absence of complete genetic data. The creation of reliable molecular tools for early diagnosis, study into the environmental factors affecting GBS prevalence, and the examination of efficient management techniques to lessen its effects on aquaculture should be the top priorities for future research initiatives. Furthermore, for a comprehensive understanding of GBS in *O. niloticus*, interdisciplinary approaches involving ecology, microbiology, and fish health management must be integrated.

## 10. Conclusions

Worldwide, *S. agalactiae* poses a major danger to *O. niloticus* aquaculture, leading to substantial mortality rates and financial losses. Given that it may spread laterally and vertically, as well as in the presence of stressors such as overcrowding, poor water quality, and management practices, its emergence as a pathogen highlights vulnerabilities in *O. niloticus* farming. In order to prevent epidemics, more widespread implications include the need for improved biosecurity measures and ecologically friendly farming practices. Future studies should focus on understanding the dynamics of the pathogen’s spread, developing effective polyvalent vaccines and bacteriophage therapy, and looking into probiotic therapies, in order to increase *O. niloticus* resistance to infections. Furthermore, a deeper comprehension of disease control tactics might be possible by looking at the environmental factors that influence *S. agalactiae* pathogenicity.

## Figures and Tables

**Table 1:** Natural isolation of *Streptococcus agalactiae* from different mass mortalities of cultured various fish species.

Host	Accession Number	Country	Reference
Silver pomfret, *Pampus argenteus*	NS	Kuwait	Duremdez et al. (2004) [50]
Nile tilapias reared in hapas nets and earth nurseries	NS	Parana State, Brazil	Salvador et al. (2005) [54]
Cultured red tilapia *Oreochromis* sp. and Nile tilapia *O. niloticus*	NS	Thailand	Suanyuk et al. (2008) [55]
Cultured Nile tilapia	EU853480-EU853508	Brazil	Mian et al. (2009) [27]
Pond cultured tilapia	GU217535, GU217531	China	Ye et al. (2011) [35]
Cage-cultured golden pompano (*Trachinotus blochii*)	EF092913	Malaysia	Amal et al. (2012) [56]
Wild giant Queensland grouper, *Epinephelus lanceolatus*	NS	Australia	Bowater et al. (2012) [40]
Cage cultured red hybrid tilapia, *Oreochromis niloticus* × *O. mossambicus*	EF092913	Malaysia	Amal et al. (2015) [57]
Cultured *O. niloticus*	NS	Columbia	Barato et al. (2015) [58]
Hybrid tilapia (*Oreochromis niloticus* × *O. aureus*)	NR117503	Saudi Arabia	Al-Harbi (2016) [59]
Hybrid tilapia	KT869025	Egypt	Laith et al. (2017) [33]
Cultured Nile tilapia	MF113267	Indonesia	Suhermanto et al. (2019) [60]
Cultured tilapia (*Oreochromis* spp.)	NS	Taiwan	Sudpraseart et al. (2021) [38]
Cultured *O. niloticus*	NS	Bangladesh	Rahman et al. (2021) [26]
Cultured *O. niloticus*	OP580171, OP580064 and OP584472	India	Preenanka et al. (2024) [39]
Cultured *O. niloticus*	MW599202	Egypt	Abdallah et al. (2024) [25]

NS: not stated.

**Table 2:** Antimicrobial susceptibility of GBS strains to main antibiotics used in fish farms.

	Antibiotic	Reference
Sensitive	Chloramphenicol, Erythromycin, Rifampicin, Ampicillin, Sulfamethoxazole/trimethoprim, Tetracycline, Oxytetracycline, Gentamicin, Ciprofloxacin, Amoxicillin/clavulanic acid, Linomycin, Cephalexin, Nitrofurantoin, Ceftiofur, Florfenicol, Penicillin, Imipenem, Ceftriaxone, and Streptomycin	Evans et al. [17], Soto et al. [41], Ali Abuseliana et al. [112], Aisyhah et al. [129], Faria et al. [130], Chideroli et al. [132].
Resistant	Rifampin, Ampicillin, Amoxicillin/clavulanic acid, Streptomycin, Kanamycin, Neomycin, Amikacin, Enrofloxacin, Ciprofloxacin, Norfloxacin, Marbofloxacin, Gentamicin, Tobramycin, Sulfamethoxazole/trimethoprim, Tetracycline, Oxytetracycline, Oxolinic acid, Florfenicol, Nitrofurantion, Penicilin, and Erythromycin	Evans et al. [17], Soto et al. [41], Ali Abuseliana et al. [112], Aisyhah et al. [129], Faria et al. [130], Chideroli et al. [132].

## Data Availability

All the data are included in the tables and figures of this article.
